# Practice of Placenta Submission for Histopathological Examination, Experience of a Teaching/Tertiary Care Hospital in Saudi Arabia

**DOI:** 10.7759/cureus.17364

**Published:** 2021-08-22

**Authors:** Hessa M Aljhdali, Layla S Abdullah, Dalia A Alhazmi, Ahmed M Almosallam, Nabeel S Bondagji

**Affiliations:** 1 Anatomic Pathology, King Abdulaziz University Faculty of Medicine, Jeddah, SAU; 2 Anatomic Pathology, King Abdulaziz University Hospital, Jeddah, SAU; 3 Anatomic Pathology, King Faisal Specialist Hospital and Research Centre, Jeddah, SAU; 4 Obstetrics and Gynecology, Fetomaternal Medicine, King Abdulaziz University Faculty of Medicine, Jeddah, SAU

**Keywords:** placenta, examination, indications, submission, guidelines

## Abstract

Objectives

The aim of this study is to determine the appropriateness of histopathologic examination of the placenta at King Abdulaziz University Hospital (KAUH), Jeddah, based on the guidelines of the College of American Pathologists (CAP).

Methods

It is a retrospective review of obstetric and pathologic records for all deliveries at KAUH, between January 1, 2017, and April 30, 2019. The placentae were assessed for eligibility to undergo pathologic examination. Furthermore, examined and non-examined placentae meeting the CAP criteria were compared based on their actual indications.

Results

There were 8,929 deliveries, of which 1,444 (16.2%) placentae met the CAP guidelines. A total of 583/1,444 placentae (40.4%; 95% confidence interval [CI] = 37.8-43) were sent for pathologic examination. Of the 7,485 placentae that did not require submission for pathological examination, as determined by the pathologist, 7,456 (99.6%; 95% CI = 99.4-99.7) were not submitted appropriately. The labor and delivery staff were more likely to submit placentae with fetal/neonatal indications rather than those with maternal indications for examination, which was statistically significant (odds ratio = 6.5; 95% CI = 5.08-8.30).

Conclusion

While most of the examined placentae at KAUH met the CAP guidelines, there was a substantial under-submission of eligible placentae. Further studies are advised to reveal the reasons behind this underestimation so that correctional measures may be adopted, as placenta examination is a valuable tool to understand the risk factors and pathogenesis of deleterious maternal, neonatal, and fetal events.

## Introduction

The placenta is a structure that provides a solid physical link between the fetus and mother [1]. Multiple and variable factors might affect the health of the placenta and its accessories during pregnancy, which, in turn, might alter the status of the embryo, host, or vice versa [2]. Based on these facts, examination of placentae is a valuable tool to understand the risk factors and pathogenesis of deleterious maternal, neonatal, and fetal events. In addition, examination of placentae helps in the prevention of these events and providing treatment, which can be offered in future pregnancies [3]. Further, it assists in handling the medico-legal issues of malpractice cases [4,5].

The study of placental specimens would provide an educational prospect; however, histopathological examination of all placentae would be impractical, especially with a large number of deliveries. The Joint Commission of Accreditation of Healthcare Organizations in the United States asserts that normal placentae from normal deliveries do not require examination or submission for pathologic evaluation [6]. The exact definition of a normal placenta is unclear; nevertheless, it is acknowledged that a placenta should be submitted for evaluation whenever this will be of clinical value [7]. Nowadays, most hospitals choose to follow protocols to select placentae that require further microscopic assessment [8]. King Abdulaziz University Hospital (KAUH) adopted the guidelines suggested by the College of American Pathologists (CAP), which were first published in 1997 [9].

The CAP guidelines mention that the selection of placentae following vaginal or caesarian delivery relies on the responsible delivering physician and midwife. Therefore, it is recommended that physicians or midwives perform a complete and dynamic evaluation of the placenta in the delivery room, with documentation of findings. Based on the gross appearance of the placenta and specific clinical indications, the decision to submit the specimen for histopathological analysis will be made [10].

The CAP guidelines include criteria for the selection of placentae. These criteria are segregated into three categories: (1) maternal, (2) fetal/neonatal, and (3) placental indications. The suggested maternal indications include systemic disorders (e.g., diabetes, impaired glucose metabolism, hypertensive disorders, collagen diseases, seizures, severe anemia [< 9 g], premature delivery (equal or < 34 gestational weeks), post-maturity (>42 gestational weeks), peripartum fever, infection, unexplained third-trimester bleeding, clinical concern for infection (syphilis, cytomegalovirus, rubella, toxoplasma, primary herpes, and human immunodeficiency virus), polyhydramnios, oligohydramnios, unexplained or recurrent pregnancy complications (stillbirth, intrauterine growth retardation, spontaneous abortion, and premature birth), abruptio placenta, invasive procedure with suspected placental injury, non-elective pregnancy termination, and thick meconium. The recommended fetal/neonatal indications include infant admitted or transferred to a non- level-one nursery, stillbirth/perinatal death, compromised clinical condition, hydrops fetalis, birth weight < 10th percentile, seizures, infection/sepsis, major congenital anomalies, discordant twin growth > 20% weight difference, multiple gestations with the same-gender twins, and fused placentae. Finally, the recommended placental indications include physical abnormalities (infarct, mass, vascular thrombosis, retroplacental hematoma, amnion nodosum, abnormal coloration or opacification, malodor), small or large placenta size or weight, umbilical cord lesions, and total umbilical cord length less than 32 cm at term [11].

These guidelines are intended as a standardized policy to be used when certain maternal, fetal, and placental conditions indicate the need for gross and microscopic interpretation of the placenta by a pathologist. Although the KAUH has adopted these guidelines, acknowledgment of the implication of eligible placenta evaluation by obstetricians, board trainees, and midwives is not completely clear.

The purpose of this study is to determine the frequency of placentae eligible for histopathologic examination, and the appropriateness of placenta submission at KAUH, according to the adopted CAP guidelines.

## Materials and methods

This study is a retrospective review of placentae from all deliveries at KAUH, Jeddah, between January 1, 2017, and April 30, 2019. It was conducted in compliance with the ethical standards of institution, and according to the principles of the Declaration of Helsinki. After taking institutional ethical committee approval, a total of 8,929 placentae were included in the study by reviewing records of all registered live-birth deliveries as well as those that ended with intra-uterine fetal demise, at or after the 20th week of gestation. However, all pregnancies that ceased with miscarriages prior to the 20th week of gestation were excluded from the study. The review was conducted in two locations: (1) Anatomic Pathology Department, and (2) Labor & Delivery Unit in Obstetrics and Gynecology Department. 

First, the pathology archives created by the anatomic pathology department were reviewed by the pathologist to determine the indication for submission of placentae, when evident. For the review, appropriate Systemized Nomenclature of Medicine (SNOMED) codes were used to search for the following parameters: date of receiving the specimen, hospital identification number, patient demographics (age and gender), clinical diagnosis, topography, and morphology information.

Subsequently, obstetric and birth history from the delivery logs and electronic medical records were reviewed, taking note of the following considerations: delivery date, gestational age, labor remarks, birth weight, neonatal health status, placenta assessment remarks, and, accordingly, presence or absence of any indication for submitting placenta for pathological evaluation according to the CAP guidelines.

Statistical analysis

Descriptive analyses were used to calculate the frequencies and percentages of eligible placentae, and to correlate placentae submitted with those that did not require submission for histopathological evaluation based on the CAP recommendations. Furthermore, the submitted and non-submitted eligible placentae were further segregated based on their primary indications, when evident, for comparison. Each case was regarded as a true or false positive based on the adopted guidelines. The sensitivity, specificity, predictive values, and prevalence with 95% confidence intervals (CIs) were calculated based on the 2 x 2 contingency table. Finally, the four categories were further analyzed for any significant association with placental submissions. All statistical analyses were performed using the IBM SPSS utility v25 program.

## Results

There were 8,929 registered deliveries from January 1, 2017, to April 30, 2019, at the KAUH in Jeddah. A review of the obstetric records revealed that 1,444 (16.2%) placentae met the CAP guidelines for placental submission for histopathologic examination, 496 (5.6%) placentae did not meet the CAP criteria but had other clinical indications, such as prelabor rupture of membranes and premature rupture of membranes. In total, 6,989/ 8,929 (78.3%) placentae did not require submission.

A total of 612 placentae were sent for pathological examination. Among these submitted placentae, 583 (95.3%) had actual indications for histopathological evaluation based on CAP recommendations, while 27 (4.4%) out of them had other clinical indications for submission. These included: premature rupture of membranes, rupture of membranes, cesarean section (C/S) due to a previous uterine scar, pregnancy on IUCD, breech, fibroid, and previous history of molar pregnancy. Furthermore, only two (0.3%) out of 612 submitted placentae were found to not meet CAP criteria for placenta examination (Table 1).

**Table 1:** Frequencies of examined vs. unexamined placentae according to recommended CAP guidelines and other clinical indications. CAP: College of American Pathologists.

	Examined placentae, N (% out of total examined)	Unexamined placentae, N (% out of total unexamined)
CAP recommended	583 (95.3)	861 (10.4)
Other clinical indications	27 (4.4)	469 (5.6)
Not recommended	2 (0.3)	6987 (84)
Total	612	8317

Among the 1,444 eligible placentae, 583 (40.4%; 95% CI = 37.8-43) were sent for pathological examination. Furthermore, 7,456 out of 7,485 non-eligible placentae (99.6%; 95% CI = 99.4-99.7), were not properly sent due to a lack of indication of submission as concluded by the reviewing pathologist. There was a moderate level of agreement among the assigned labor /delivery staff, and pathologist, with respect to the eligibility of placentae submission for histopathological examination based on adopted criteria, with a kappa coefficient (κ) = 0.52 (95% CI = 0.50-0.55) (Table 2).

**Table 2:** Level of agreement for handling placentae as per the CAP guidelines. CAP: College of American Pathologists.

	Did the placenta meet the CAP criteria	
Placenta submitted	Yes	No
Yes	583	29
No	861	7456
*Positive predictive value (PPV) = 95.3%		
*Negative predictive value (NPV) = 89.6%		
*Sensitivity = 40.4%		
*Specificity = 99.6%		

All 612 (6.9%) examined vs. 8,317 (93.1%) non-examined placentae were further classified based on CAP indications, as follows: (none, maternal, fetal/neonatal, and placental). The data showed that 321 (52.5%) of the examined placentae had fetal/neonatal indications, 185 (30.2%) had maternal indications, and 77 (12.6 %) had placental indications. On the other hand, the majority of the eligible placentae that were not examined had maternal indication, with a total of 662 (8%) (Table 3).

**Table 3:** Association between CAP indications, and whether the placentae were submitted for examination. CAP: College of American Pathologists.

Indication	Submitted Placentae, N (% out of total submitted)	Non-submitted placentae, N (% out of total non-submitted)
None	29 (4.7)	7456 (89.6)
Maternal	185 (30.2)	662 (8)
Fetal/neonatal	321 (52.5)	177 (2.1)
Placental	77 (12.6)	22 (0.3)

Further analysis showed a significant association between the mentioned CAP indications, regardless of the histopathological evaluation status of the placentae (P < 0.001). Moreover, the assigned labor and delivery staff were more likely to send placentae with fetal/neonatal indications rather than those with maternal indications and this difference was statistically significant (odds ratio [OR] = 6.5; 95% confidence interval [CI] = 5.08-8.30).

## Discussion

The significance of placenta evaluation has sparked the discussion of several variable studies debating whether proper histopathological examination can predict undesirable perinatal and pregnancy outcomes [12-15]. This information can be used to enhance patient counseling with regard to future pregnancies and life-long health [16]. Assembling the data from placental pathology can be achieved by the optimal practice of eligible placenta submission based on the guidelines adopted by the institution [4,17]. Interestingly, the rate of submission of eligible placenta can be used as a means to assess the comprehension of labor and delivery staff regarding the indication for placenta evaluation. It also assesses the level of agreement with policy adopted by the institution. In general, the obstetricians, midwives, and trainees at King Abdulaziz University Hospital demonstrated a positive attitude toward the adopted CAP guidelines. However, to the best of our knowledge there is no published study in the literature; that reflects on the practice of placenta examination in Saudi Arabian hospitals according to institutional recommendations.

In this study, we studied 8,929 deliveries within a period of two years and four months. Of these deliveries, 16.2% had one or more indications for placental examination, indicating that almost one-sixth of the delivered placentae in our institution were eligible for histopathological evaluation based on the CAP criteria. In addition, 583 (95.3%) out of 612 placentae sent to the pathology laboratory were appropriately submitted for histopathologic examination, while 27 (4.4%) had other clinical indications. Only two (0.3%) placentae were not appropriately sent for unclear reasons. The lack of over-submission of non-eligible placentae gives the impression that the concerned team is agreeable to following the adopted CAP recommendations. However, 861 (9.6%) of the 8,929 deliveries were not sent for evaluation when they were supposed to. The underestimation of these placentae can be attributed to the lack of awareness of assigned labor and delivery staff regarding CAP guidelines, lack of communication between team members, misunderstanding of ambiguous pathologic terminology related to clinical diagnoses, or, less likely, disagreement with the CAP submission criteria [7,18].

Interestingly, the literature review showed variable detection rates for eligible placentae among different institutions, most of which had their share of revising a number of placentae that were not sent for examination when they supposed to. For example, a retrospective study in the USA by Aysha and Rafaat published in 2020, showed that 213 (42.6%) out of 500 placentae should have been submitted for pathology evaluation and only 135 (27% of the total) were submitted [19]. Also, Amber et al.’s retrospective study, conducted at a university hospital in the United States in 2010, discovered that 757 (56.2%) out of approximately 1,346 delivered placentae had indications for pathologic examination according to CAP guidelines; however, 575 (81.8%) of the eligible placentae were actually examined [20]. Moreover, Al Harazi and Frass et al. in Yemen in 2007 reviewed the records of 11,472 deliveries and found that although 1,501 (13.1%) met CAP guidelines for placental submission, only 73 (4.9%) of these were examined [21]. In addition, Spencer et al.’s research group in Australia published an article in 2003. The article argued that 49.5% of all deliveries studied within the three-month period met the CAP guidelines for placental examination; however, the actual placental examination occurred in only 17.8% of the deliveries. Only 1.1% of placentae that did not meet CAP guidelines were examined [22]. Curtin et al. study showed a similar finding at Strong Memorial Hospital, where only 18.2% out of 1,360 deliveries had a placental examination [23]. The findings of these studies are similar to those of ours, in which the majority of the placenta sent for pathological evaluation met the CAP recommendations. Nevertheless, the debate was on a significant number of placentae that were not submitted for pathological studies, even though they met the CAP criteria.

In our study, we also found that 321 (52.5%) of the submitted placentae had fetal indication, while 662 (8%) of the eligible placenta that was not evaluated had maternal indication. Additionally, the comparison between the CAP indications point to a statistical significance where the assigned staff were more likely to send placentae with fetal/neonatal indications than those with maternal indications (odds ratio [OR] = 6.49; 95% confidence interval [CI] = 5.08-8.30). The data concur with the findings exhibited by Amber et al. and Curtin et al. [20,23]. This outcome suggests that obstetricians are more liable to acknowledge fetal/neonatal indications among others during their practice for some reason.

Implementing the guidelines of the CAP is the best way to ensure correct evaluation of all eligible placentae and to avoid redundancy. The lack of consistency between the expected and observed placental examinations might suggest policy evaluation at our institution. In addition, adequate training for new obstetricians, board trainees, midwives, and pathologists might resolve the issue of inadequate submission for placental examination.

With regard to our study limitations, we considered that it would be more appropriate for a perinatal pathologist instead of a general pathologist to study the eligibility of placenta submission for examination. Unfortunately, we do not currently have a specialized perinatal pathologist in the western region of the kingdom. Furthermore, during our research, we faced some limitations in collecting data from the labor records because of the lack of digitized documentation. Digitized storage system for labor record is recommended to facilitate the conduction of further studies in the future.

## Conclusions

The majority of the placentae sent for microscopic examination at KAUH met the CAP guidelines. However, there was a slight under-submission of eligible placentae for unknown reasons. Measures to ascertain the exact reasons for the underestimation of placentae for examination should be undertaken. It is recommended to reevaluate the policy, and use a routine checklist in future to increase the rate of placenta submission correctly.
